# HBeAg-positive patients with HBsAg  < 100 IU/mL and negative HBV RNA have lower risk of virological relapse after nucleos(t)ide analogues cessation

**DOI:** 10.1007/s00535-021-01812-0

**Published:** 2021-07-22

**Authors:** Yandi Xie, Minghui Li, Xiaojuan Ou, Sujun Zheng, Yinjie Gao, Xiaoyuan Xu, Ying Yang, Anlin Ma, Jia Li, Yuan Huang, Yuemin Nan, Huanwei Zheng, Bo Feng

**Affiliations:** 1 Beijing Key Laboratory of Hepatitis C and Immunotherapy for Liver Diseases, Beijing International Cooperation Base for Science and Technology On NAFLD Diagnosis, Peking University Hepatology Institute, Peking University People’s Hospital, Beijing, 100044 China; 2 Department of Hepatology Division, Beijing Ditan Hospital, Capital Medical University, Beijing, 100015 China; 3 Liver Research Center, Beijing Friendship Hospital, Capital Medical University, Beijing, 100050 China; 4 Complicated Liver Diseases and Artificial Liver Treatment and Training Center, Beijing Municipal Key Laboratory of Liver Failure and Artificial Liver Treatment and Research, Beijing Youan Hospital, Capital Medical University, Beijing, 100069 China; 5 The Fifth Medical Center, Department of Infectious Diseases, General Hospital of PLA, Beijing, 100039 China; 6 Department of Infectious Diseases, Peking University First Hospital, Beijing, 100034 China; 7 Department of Infectious Diseases, The Second Hospital of Xingtai, Xingtai, 054001 China; 8 Department of Infectious Disease, China-Japan Friendship Hospital, Beijing,, 100029 China; 9 Department of Liver Disease, Tianjin Second People’s Hospital, Tianjin, 300192 China; 10 Department of Hepatopancreatobiliary Disease, School of Clinical Medicine, Beijing Tsinghua Changgung Hospital, Tsinghua University, Beijing, 102218 China; 11 Department of Traditional and Western Medical Hepatology, The Third Hospital of Hebei Medical University, Shijiazhuang, 050051 China; 12 Department of Liver Disease, Shijiazhuang Fifth Hospital, Shijiazhuang, 050021 China

**Keywords:** Cessation, HBV RNA, HBcrAg, Virological relapse, HBsAg loss

## Abstract

**Background:**

Nucleos(t)ide analogues (NAs) cessation is not widely practiced and remains a controversial, but highly relevant subject in patients infected with hepatitis B virus (HBV). We aimed to explore the related factors for safe NAs cessation.

**Methods:**

This is a multicenter prospective cohort study. Overall, 139 initially HBV e antigen (HBeAg)-positive patients meeting the stopping criteria were included in 12 hospitals in China. Enrolled patients ceased NAs and were followed up every 3 months for 24 months or until clinical relapse (CR).

**Results:**

The 24 month cumulative rates of virological relapse (VR), CR, HBeAg reversion and HBV surface antigen (HBsAg) loss were 50.4, 24.5, 11.5 and 9.4%, respectively. Patients with end of treatment (EOT) HBsAg  < 100 IU/mL plus negative HBV RNA had the lowest 24 month cumulative VR rate (5 vs 58%, *p* < 0.001). EOT HBsAg  ≥ 2 log_10_ IU/mL [odds ratio (OR) = 6.686*, p* = 0.006], EOT positive HBV RNA (OR = 3.453*, p* = 0.008) and EOT hepatitis B core-related antigen (HBcrAg)  ≥ 4log U/mL (OR = 3.702*, p* = 0.002) were found to independently predict the risk of VR. To predict VR, the area under the receiver-operating characteristic (AUROC) value of the EOT HBsAg  < 100 IU/mL plus EOT HBV RNA negative was 0.698 (*p* < 0.001), which was higher than other parameters alone or combinations.

**Conclusions:**

NAs cessation is suitable only for a small and selected patients. An EOT HBsAg  < 100 IU/mL and EOT negative HBV RNA identified a patient with low risk of off-treatment VR.

## Introduction

Long-term nucleos(t)ide analogue (NAs) treatment has been proven to delay disease progression in patients infected with hepatitis B virus (HBV) [1]. As viral polymerase inhibitors, NAs inhibit only viral replication, but they could not completely eradicate the stably existing covalently closed circular DNA (cccDNA) [2]. Therefore, life-long NAs treatment is necessary, but causes a financial burden and potential drug toxicity [3]. NAs withdrawal remains controversial. Some studies suggested high rates of virological relapse (VR) after NAs cessation [4, 5], whereas other studies found sustained virological response and hepatitis B surface antigen (HBsAg) loss in some patients who ceased NAs [6, 7]. Therefore, it is meaningful to identify factors associated with safe NAs discontinuation.

Some factors associated with relapse after NAs cessation have been reported, including baseline HBV DNA level, HBV genotype [8], treatment and consolidation duration [9] and end-of-treatment (EOT) HBsAg level [8]. Some studies found that serum HBsAg loss rate was increased significantly after NAs cessation [10, 11]. A low serum quantitative EOT HBsAg level  < 100 IU/mL, recently proposed for possible NAs treatment cessation in both HBeAg-positive and HBeAg-negative chronic hepatitis B (CHB) patients [12, 13]. Long-term NAs treatment can suppress transcriptional activity of cccDNA, but a dichotomous separation of HBV DNA and HBsAg levels occurs. HBsAg may correlate with intrahepatic cccDNA, but is encoded both by cccDNA and from integrated viral genomes.

Serum HBV RNA transcribed from cccDNA and presented as encapsidated virion-containing pregenomic RNA (pgRNA) [15]. Although HBV is an enveloped DNA virus, the serum viral population contain virion-like particles containing HBV RNA and an empty viral envelope containing capsid without genomes [16]. Therefore, serum HBV RNA is another considerable biomarker for cccDNA activity. Since serum HBV RNA can be quantified recently, few studies have researched the association between EOT HBV RNA level and relapse after NAs cessation [17, 18].

Serum hepatitis B core-related antigen (HBcrAg), including the hepatitis B core antigen, hepatitis B e antigen (HBeAg) and the 22 kd precore protein, has been found as another potential marker because of its association with cccDNA level [19]. Previous studies suggested that serum HBcrAg level had predictive value for sustained response to NAs treatment, HBeAg seroconversion, the risk of hepatocellular carcinoma (HCC) development [19]. However, whether serum HBcrAg alone or combining HBV RNA and HBcrAg could predictive relapse after NAs cessation is still unclear.

In this study, we established a multicenter prospective cohort of initially HBeAg-positive CHB patients who discontinued NAs treatment, to observe the relapse and HBsAg loss rates and explore the related factors, especially the EOT HBsAg, HBV RNA and HBcrAg levels, for successful NAs cessation.

## Methods

### Patients

Initially HBeAg-positive CHB patients who met the stopping criteria ceased NAs and were followed up from January 2017 to December 2020 in 12 hospitals of Beijing, Tianjin and Hebei province in China. The stopping criteria was defined as undetectable serum HBV DNA, normal serum alanine aminotransferase (ALT) levels and HBeAg seroconversion for at least 3 years, meanwhile, the NAs therapy duration more than 4 years, according to the Chinese guidelines of prevention and treatment for chronic hepatitis B [20].

Patients with cirrhosis, HCC, human immunodeficiency virus coinfection, hepatitis C virus coinfection, autoimmune liver diseases, genetic metabolic liver disease, chronic alcoholism and history of immunosuppressive therapy or organ transplantation were excluded.

Enrolled patients were followed up with clinical and laboratory assessments every 3 months for 24 months after NAs cessation or until clinical relapse (CR). In case of VR or CR, more frequent ALT and HBV DNA assay(s) were performed. Retreatment was started if clinical relapse was observed.

This is a multicenter prospective cohort study, which was registered in the system of Chinese Clinical Trail Registry (registration number: ChiCTR1900020836). The study was approved by the Institutional Review Board of Peking University People’s Hospital (2017PHB001‐01). Written informed consent was obtained from all patients.

### Definitions

VR was defined as an HBV DNA  > 2000 IU/mL. CR was defined as VR plus ALT  > 2 ULN. Consolidation therapy was defined as duration of treatment after the first report of HBeAg seroconversion and lasted until NAs cessation. Liver cirrhosis was diagnosed by ultrasonography, computerised tomography or Magnetic Resonance Imaging findings and supplemented with the decompensation presence of ascites, splenomegaly, varices and/or thrombocytopenia.

### Serological and virological test

Serum ALT levels were tested at local laboratories with the ULN of 40 U/L. HBV DNA, HBsAg were assessed in the central laboratory located in Peking University People’s Hospital. HBV DNA was assayed using the Roche COBAS TaqMan HBV test with a lower detection limit of 20 IU/mL. Serum HBsAg was quantified by the Architect I2000SR (Abbott) with the range of 0.05–250 IU/mL. If the HBsAg level was  > 250 IU/mL, serial 1:100–1:1000 dilution was performed.

### Serum HBV RNA assay

HBV RNA was isolated from 200 μL serum using the Diagnostic kit for Hepatitis B virus pgRNA (PCR-fluorescence probing) (Hotgen Biotech, Beijing, China) according to the manufacturer’s protocol and treated with DNase I. Isolated HBV RNA was reverse transcribed and detected by quantitative real-time polymerase chain reaction in ABI Prism 7500 Real-time PCR System (ABI, USA). The results are presented as copies/mL.

### Serum HBcrAg assay

The serum HBcrAg level was tested using a chemiluminescent enzyme immunoassay (Lumipulse G HBcrAg assay) by Lumipulse G1200 analyser (Fujirebio, Japan), with a lower limit of quantification of 3 logU/mL. If samples with concentration  > 7 logU/mL, dilution and retest were performed.

### Statistical analysis

The data are expressed as median (interquartile range) for continuous variables and as numbers (percentages) for categorical variables. The HBsAg, HBV DNA and HBV RNA levels were logarithmically transformed for statistical analysis. Differences between groups were analysed using the *χ*^2^ test or the student’s *t* test. The cumulative rates of VR, CR, HBeAg reversion and HBsAg loss were calculated using Kaplan–Meier analyses and compared by the log-rank test. Multivariable logistic regression analysis was used to assess predictors of off-treatment relapse and HBsAg loss. The correlation between two variables was compared using the regression and Pearson’s correlation coefficients (*r*). The statistical analysis was conducted using IBM SPSS software version 26.0 and GraphPad Prism 7.0 software. A two-tailed *p* value < 0.05 was considered statistically significant.

## Results

A total of 139 initially HBeAg-positive CHB patients without cirrhosis treated with NAs for at least 4 years and who had HBeAg seroconversion for at least 3 years were included in this study. They all discontinued NAs when attended this study and were followed up each 3 months for 24 months. During follow-up, none of the patients experienced hepatic decompensation, cirrhosis, HCC or died.

### Clinical and serological characteristics at end of treatment

All patients (58.3% male, median age, 36 years) were treated for a duration of 6.4 (4.7–8.6) years with NAs, and most of them were treated with entecavir (71.2%). The duration of undetectable HBV DNA and HBeAg seroconversion before end of treatment was 5.8 (4.3–7.8) years and 4 (3.5–5.8) years, respectively. All subjects had detectable serum HBsAg in a range from 0.05 to 18,512.6 IU/mL, with 22 (15.8%) patients  < 100 IU/mL and 39 (28.1%) patients between 100 and 1000 IU/mL. Significant differences of the EOT HBsAg and HBV RNA levels were observed between patients with VR and non-VR. When comparing with VR, those without VR had lower EOT HBsAg levels (median 3.4 vs 2.9 log_10_ IU/mL, *p* < 0.001) and HBcrAg levels (median 4 vs 3.5 logU/mL, *p* < 0.001), simultaneously, more frequently negative HBV RNA (55.7 vs 87%, *p* < 0.001). Clinical and serological characteristics of patients before antiviral treatment initiation and at end of treatment are shown in Table 1.

**Table 1:** Characteristics of patients at start of treatment, end of treatment and after withdrawal

	All (*n* = 139)	VR (*n* = 70)	Non-VR (*n* = 69)	*p* value
Start of treatment				
HBV DNA, log_10_ IU/mL	5.9 (5.4–6.8)	5.8 (5–6.7)	5.9 (5.4–6.9)	0.779
HBsAg, log_10_ IU/mL	3.5 (3.1–3.8)	3.5 (3.1–3.8)	3.5 (3.1–4.1)	0.494
Family history of HBsAg positive	70 (50.4%)	37 (52.9%)	33 (47.8%)	0.553
Family history of HCC	13 (9.4%)	9 (12.9%)	4 (5.8%)	0.313
End of treatment				
Age, y	36 (31–45)	36 (32–41.3)	37 (31–46.5)	0.773
Male gender	81 (58.3%)	34 (48.6%)	47 (68.1%)	0.019
Body mass index, kg/m^2^	23 (21.1–24.8)	22.7 (21–24.8)	23.4 (21.6–25.1)	0.298
Current antiviral treatment				0.182
Entecavir	99 (71.2%)	53 (75.7%)	46 (66.7%)	
Tenofovir	16 (11.5%)	9 (12.9%)	7 (10.1%)	
Others	24 (17.3%)	8 (11.4%)	16 (23.2%)	
Treatment duration,y	6.4 (4.7–8.6)	6 (4.7–8.7)	6.8 (4.4–8.8)	0.502
Undetectable HBV DNA duration,y	5.8 (4.3–7.8)	5.7 (4.3–7.7)	5.8 (4.5–7.9)	0.435
HBeAg seroconversion duration, y	4 (3.5–5.8)	3.9 (3.5–5.6)	4.3 (3.5–6.2)	0.524
Liver stiffness, kPa	4.8 (4.1–5.8)	4.6 (3.9–5.9)	4.8 (4.2–5.6)	0.409
CAP, dB/m	218 (191–258)	211 (180.5–242.5)	234 (194–262.8)	0.284
HBsAg, log_10_ IU/mL	3.2 (2.6–3.6)	3.4 (2.9–3.7)	2.9 (2–3.3)	< 0.001
< 2 log_10_ IU/mL	22 (15.8%)	3 (4.3%)	19 (27.5%)	< 0.001
HBV RNA,log_10_ copies/mL	0 (0–2)	0 (0–2.5)	0 (0–0)	< 0.001
Negative HBV RNA	99 (71.2%)	39 (55.7%)	60 (87%)	< 0.001
HBcrAg, logU/mL	3.8 (3.3–4.2)	4 (3.6–4.3)	3.5 (3–3.9)	< 0.001
< 4 logU/mL	88 (63.3%)	32 (45.7%)	56 (81.2%)	< 0.001
After withdrawal				
6 months after withdrawal				
HBsAg, log_10_ IU/mL	3.2 (2.5–3.6)	3.5 (3–3.7)	2.9 (1.7–3.3)	< 0.001
HBV RNA, log_10_ copies/mL	0 (0–2.4)	2.4 (0–3.5)	0 (0–0)	0.015
HBcrAg, log U/mL	3.7 (3.3–4.3)	4.2 (3.6–4.8)	3.4 (2.8–3.8)	0.001
12 months after withdrawal				
HBsAg, log_10_ IU/mL	3 (2.4–3.5)	3.3 (2.9–3.6)	2.8 (1.7–3.3)	0.001
HBV RNA, log_10_ copies/mL	0 (0–2.4)	2.3 (0–3.6)	0 (0–0)	0.019
HBcrAg, logU/mL	3.6 (3.0–4.1)	4 (3.5–5)	3.2 (2.7–3.6)	< 0.001
24 months after withdrawal				
HBsAg, log_10_ IU/mL	2.9 (2.1–3.3)	3.1 (2.7–3.5)	2.7 (1.6–3.2)	0.015
HBV RNA, log_10_ copies/mL	0 (0–1.9)	1.9 (0–2.7)	0 (0–0)	0.006
HBcrAg, log U/mL	3.4 (2.7–3.9)	3.9 (3.3–4.2)	3.2 (2.5–3.6)	0.001

The data are expressed as median values (interquartile ranges) or no. (%) of individuals

*VR* virological relapse, *HCC* hepatocellular carcinoma, *CAP* the controlled attenuation parameter

### VR, CR and HBeAg reversion after NAs cessation

After NAs cessation, patients were followed up for 12 (9–24) months. The 12 month cumulative rates of VR, CR and HBeAg reversion were 38.8, 15.1 and 8.6%, respectively, and the corresponding 24 month cumulative rates were 50.4, 24.5 and 11.5%, respectively (Table 2).

**Table 2:** VR, CR, HBeAg seroconversion and HBsAg loss in patients after NAs cessation

	3 months (%)	6 months (%)	9 months (%)	12 months (%)	15 months (%)	18 months (%)	21 month (%)	24 month (%)	In total (%)
VR	9 (6.5)	21 (21.6)	15 (32.4)	9 (38.8)	5 (42.4)	4 (45.3)	2 (46.8)	5 (50.4)	70 (50.4)
CR	2 (1.4)	6 (5.8)	9 (12.2)	4 (15.1)	5 (18.7)	5 (22.3)	3 (24.5)	0 (24.5)	34 (24.5)
HBeAg reversion	1 (1)	6 (5)	3 (7.2)	2 (8.6)	1 (9.4)	1 (10.1)	0 (10.1)	2 (11.5)	16 (11.5)
HBsAg loss	2 (1.4)	2 (2.9)	0 (2.9)	2 (4.3)	2 (5.8)	1 (6.5)	1 (7.2)	3 (9.4)	13 (9.4)

The data are expressed as number of new cases per visit (cumulative incidence)

*VR* virological relapse, *CR* clinical relapse

The majority of VR and CR occurred within 12 months, especially between 6 and 9 months after NAs cessation (Table 2). The median level of elevated HBV DNA was 4.8 (3.8–6.6) log_10_ IU/mL. HBV DNA was transiently elevated in 29 (20.9%) patients (data not shown). The median level of elevated ALT was 247 (172–484) U/L. There are 36 patients only developed VR without subsequently CR. Patients with VR only had significantly more frequent of EOT HBsAg  < 100 IU/mL (5.6 vs 2.9%, *p* = 0.017) and EOT negative HBV RNA (23.5 vs 8.3%, *p* = 0.021) than patients with both VR and CR (data not shown).

**Fig. 1:**
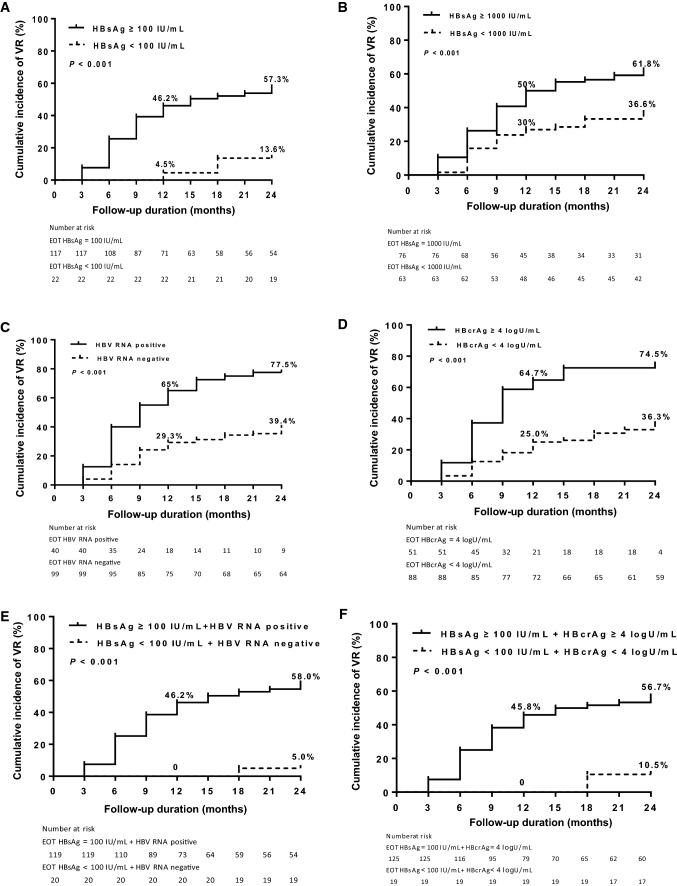

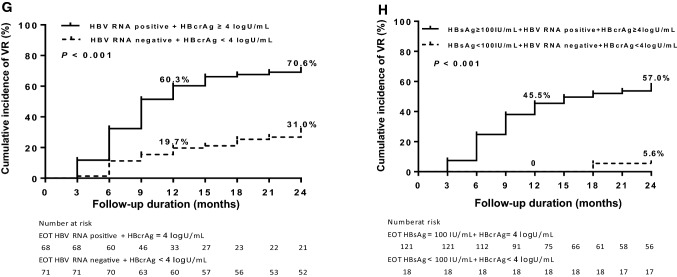
Cumulative incidences of virological relapse (VR) stratified by end of treatment (EOT) hepatitis B surface antigen (HBsAg), HBV RNA and hepatitis B core-related antigen (HBcrAg). **A** and **B** 12 month and 24 month off-therapy VR in patients by EOT HBsAg titer. **C** 12 month and 24 month off-therapy VR in patients by EOT HBV RNA status. **D** 12 month and 24 month off-therapy VR in patients by EOT HBcrAg titer. **E** 12 month and 24 month off-therapy VR in patients by EOT HBsAg titer and HBV RNA status. **F**, 12 month and 24 month off-therapy VR in patients by EOT HBsAg and HBcrAg titers. **G** 12 month and 24 month off-therapy VR in patients by EOT HBV RNA status and HBcrAg titer. **H**, 12 month and 24 month off-therapy VR in patients by EOT HBV RNA status, HBsAg and HBcrAg titers

Cumulative rates of VR stratified by EOT HBsAg, HBV RNA or HBcrAg levels are depicted in Fig. 1. Up to 24 months, cumulative incidence of VR in EOT HBsAg  < 100 IU/mL and  ≥ 100 IU/mL were 13.6 and 57.3%, respectively (*p* < 0.001, Fig. 1A). Cumulative rates of VR as stratified by EOT HBsAg  < 1000 IU/mL and  ≥ 1000 IU/mL were 36.6 and 61.8%, respectively (*p* < 0.001, Fig. 1B). Patients with negative EOT HBV RNA had significantly lower rate of VR (39.4 vs 77.5%, *p* < 0.001, Fig. 1C). The cumulative incidence of VR was significantly lower in patients with lower HBcrAg levels (< 4 logU/mL) than in those with higher HBcrAg levels (≥ 4 logU/mL) (36.3 vs 74.5%, *p* < 0.001, Fig. 1D).

When compared with each variable alone, combining these three factors could have better predictive ability. Patients with EOT HBsAg  < 100 IU/mL plus EOT negative HBV RNA had the lowest 24 month cumulative VR rate (5 vs 58%, *p* < 0.001, Fig. 1E). EOT HBsAg  < 100 IU/mL with EOT HBcrAg  < 4 logU/mL demonstrated 24 month cumulative VR rate of 10.5%, significantly lower than the remaining patients (56.7%) (*p* < 0.001, Fig. 1F). Patients with EOT negative HBV RNA plus EOT HBcrAg  < 4 logU/mL also has a decrease 24 month cumulative incidence of VR (31 vs 70.6%, *p* < 0.001, Fig. 1G). Combining the EOT HBsAg  < 100 IU/mL, EOT negative HBV RNA and EOT HBcrAg  < 4 logU/mL demonstrated 24 month cumulative rate of 5.6% (Fig. 1H), not showed obvious superiority for EOT HBsAg  < 100 IU/mL plus EOT negative HBV RNA (5%).

### Correlation between serum HBV markers in patients with VR

**Fig. 2:**
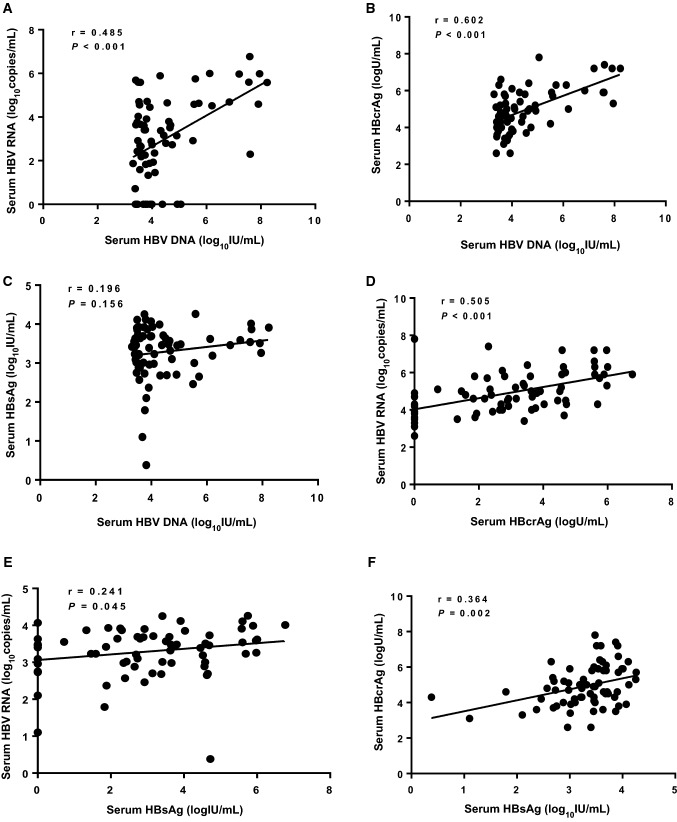
Correlation between serum hepatitis B virus (HBV) markers in patients with virological relapse. **A** Correlation between HBV RNA and HBV DNA. **B** Correlation between hepatitis B core-related antigen (HBcrAg) and HBV DNA. **C** Correlation between hepatitis B surface antigen (HBsAg) and HBV DNA. **D** Correlation between HBV RNA and HBcrAg. **E** Correlation between HBV RNA and HBsAg. **F** Correlation between HBcrAg and HBsAg

Regression and Pearson’s correlation coefficients (*r*) were used to evaluate the correlation among serum HBsAg, HBV RNA, HBcrAg and HBV DNA in patients with VR after NAs cessation. As shown in Fig. 2, there were positive correlations between serum HBV DNA levels with HBV RNA (*r* = 0.485, *p* < 0.001, Fig. 2A) and HBcrAg (*r* = 0.602, *p* < 0.001, Fig. 2B). Serum HBV DNA and HBsAg did not have good relevancy (*r* = 0.196, *p* = 0.156, Fig. 2C). In addition, positive correlation between serum HBV RNA and HBcrAg was found (*r* = 0.505, *p* < 0.001, Fig. 2D). Serum HBsAg titer had a very weak correlation with both serum HBV RNA and HBcrAg titers (*r* = 0.157 and 0.123, respectively, Fig. 2E, F).

### Changes in HBsAg after NAs cessation

**Fig. 3:**
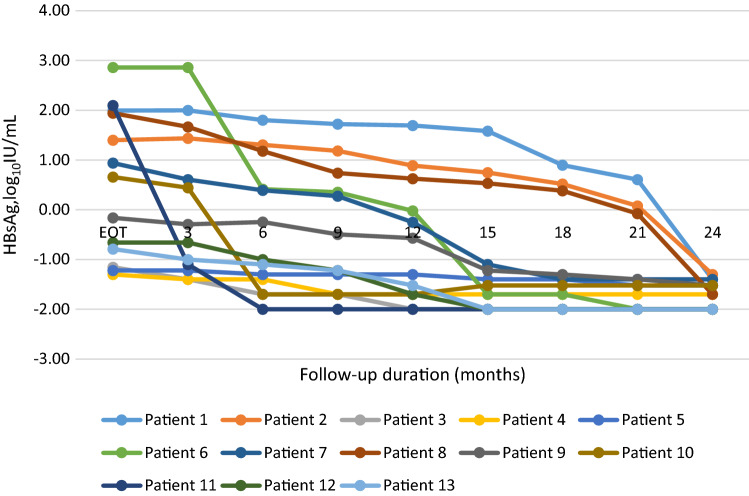
Serial hepatitis B surface antigen (HBsAg) levels in patients with HBsAg loss during follow-up after end of treatment (EOT)

The 12 month and 24 month cumulative rates of HBsAg loss were 4.3 and 9.4%, respectively (Table 2). Among these 13 (9.4%) patients achieved HBsAg loss during the follow up, 6 (46.2%) patients with EOT HBsAg  < 1 mIU/mL, 2 (15.4%) patients with EOT HBsAg between 1 and 10 mIU/mL, 3 (23.1%) patients with EOT HBsAg between 10 and 100 mIU/mL and 2 (15.4%) patients with EOT HBsAg  > 100 mIU/mL. Only 1 patient had CR at 3 months with HBsAg decreased significantly and then subsequently had HBsAg loss at 6 months after NAs cessation. The other 12 patients with HBsAg loss did not have VR or CR. Except these 13 patients with HBsAg loss, there are 5 (3.6%) patients had a decline in HBsAg of  > 1 log_10_ IU/mL after NA cessation (Fig. 3).

### Predictors for VR, CR and HBsAg loss

To identify predictors for VR, CR and HBsAg loss, the univariable and multivariable logistic regression analysis were conducted. Neither of the treatment duration and the duration of consolidation treatment were associated with VR, CR or HBsAg loss. The following three variables were found to be independently significant in predicting the risk of VR: EOT HBsAg  ≥ 2 log_10_IU/mL [odds ratio (OR) 6.686, 95% confidence interval (CI) 1.703–26.255, *p* = 0.006], EOT HBV RNA positive [OR 3.453, 95% CI, 1.387–8.597, *p* = 0.008] and EOT HBcrAg  ≥ 4 logU/mL (OR 3.702, 95% CI 1.614–8.488, *p* = 0.002). In addition, EOT HBV RNA positive (OR 4.782, 95% CI 1.968–11.621, *p* = 0.001) was associated with higher risk of CR. Age  ≥ 40 years (OR 0.867, 95% CI 0.809–0.928, *p* < 0.001) and EOT HBsAg  < 2 log_10_ IU/mL (OR 0.025, 95% CI 0.005–0.134, *p* < 0.001) were also independent predictors for HBsAg loss (Table 3).

**Table 3:** Predictors for VR, CR and HBsAg loss in patients by multivariable logistic regression

Variable	VR		CR		HBsAg loss	
	OR (95% CI)	*p* value	OR (95% CI)	*p* value	OR (95% CI)	*p* value
Age (< 40 vs ≥ 40 y)	2.218 (0.812–5.581)	0.125	1.640 (0.225–4.818)	0.402	0.867 (0.809–0.928)	< 0.001
Gender (male vs female)	1.827 (0.795–4.197)	0.156	2.203 (0.797–6.092)	0.128	0.172 (0.028–1.039)	0.050
Treatment duration (< 6 vs ≥ 6 y)	1.001 (0.984–1.019)	0.884	1.006 (0.982–1.030)	0.636	1.005 (0.976–1.035)	0.721
Consolidation treatment duration (< 5 vs ≥ 5 y)	1.994 (0.970–4.019)	0.652	0.988 (0.957–1.019)	0.435	0.996 (0.958–1.036)	0.843
HBV DNA before antiviral treatment initiation, log_10_ IU/mL	1.367 (0.952–1.963)	0.091	1.314 (0.845–2.046)	0.226	0.886 (0.466–1.683)	0.712
EOT HBsAg (≥ 2 vs < 2 log_10_ IU/mL)	6.686 (1.703–26.255)	0.006	4.537 (0.542–38.002)	0.163	0.025 (0.005–0.134)	< 0.001
EOT HBV RNA (positive vs negative)	3.453 (1.387–8.597)	0.008	4.782 (1.968–11.621)	0.001	0.416 (0.036–4.827)	0.483
EOT HBcrAg (≥ 4 vs < 4 log U/mL)	3.702 (1.614–8.488)	0.002	2.230 (0.932–5.331)	0.071	0.856 (0.117–6.237)	0.878

Note: consolidation treatment duration was defined as the treatment duration after achieving hepatitis B e antigen seroconversion

*VR* virological relapse, *CR* clinical relapse, *EOT* end of treatment, *OR* odds ratio, 95% *CI* confidence interval

**Fig. 4:**
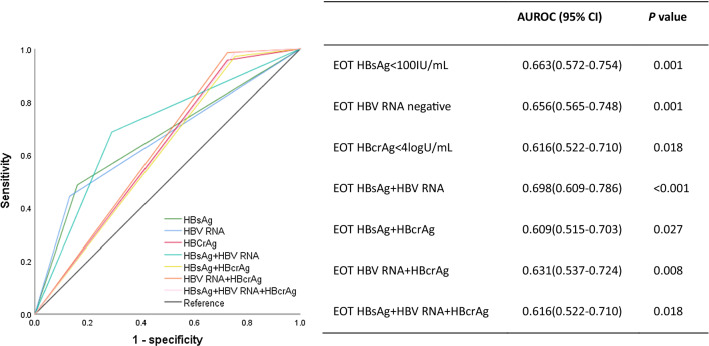
Area under the receiver-operating characteristic curves (AUROC) of end of treatment (EOT) hepatitis B surface antigen (HBsAg), hepatitis B virus (HBV) RNA, hepatitis B core-related antigen (HBcrAg) and their combination for predicting virological relapse in the cohort

To further assess the value of EOT HBsAg, HBV RNA, HBcrAg and their combination in predicting VR, the area under the receiver-operating characteristic (AUROC) values of each parameter were calculated. The results showed that the AUROC value of the EOT HBsAg  < 100 IU/mL plus EOT HBV RNA negative was 0.698 (*p* < 0.001), which was higher than other parameters alone or combinations (HBV RNA 0.656, *p* = 0.001, HBcrAg 0.616, *p* = 0.018, HBsAg plus HBcrAg 0.609, *p* = 0.027, HBV RNA plus HBcrAg 0.631, *p* = 0.008, HBsAg plus HBV RNA plus HBcrAg 0.616, *p* = 0.018, Fig. 4).

## Discussion

This prospective multicenter cohort study, including initially HBeAg-positive CHB patients who discontinued NAs treatment, found that the 12 month cumulative rates of VR, CR, HBeAg reversion and HBsAg loss were 38.8, 15.1, 8.6 and 4.3%, respectively, and the corresponding 24 month cumulative rates were 50.4, 24.5, 11.5 and 9.4%, respectively. We explored the predicting factors for safely off-treatment, especially the EOT HBsAg, HBV RNA and HBcrAg. The results showed that the combination of EOT HBsAg and HBV RNA was better for successful NAs cessation.

In our study, after a median treatment duration of 6.4 years and consolidation treatment duration of 4 years, half of patients experienced VR within 24 months after NAs cessation. The majority of VR and CR occurred within 12 months, especially between 6 and 9 months after NAs cessation. On the other hand, 4.3 and 9.4% of patients had HBsAg loss at the 12 and 24 months after end of treatment, respectively. These results suggested that safely NAs cessation might be feasible in some patients, which need a close monitoring, especially the first 12 months, during EOT follow-up. Remarkable variations of VR rates across previous studies were found, since variations of the study design (prospective or retrospective), NAs stopping rule, duration of NAs treatment and off-NAs follow-up, duration of virological response before NAs cessation, definition of VR, and other clinical factors.

Because of the stability of cccDNA in the hepatic nuclei and the difficulty of eradicating it by NAs, a high rate of VR was observed in patients after NAs cessation. Identifying the useful factors to predict VR after NAs cessation remained to be a challenge for CHB patients management. In previous studies, some factors were associated with VR after treatment cessation, including age, gender, baseline ALT, baseline HBV DNA, treatment duration and consolidated therapy duration [8, 11, 21, 22]. However, the results of this study showed no relationship between these factors and EOT VR. Obviously, there are some discrepancies among current studies, as to these parameters are not practical for determining when to stop antiviral therapy.

Considering the correlation between serum HBV RNA and intrahepatic pregenomic RNA [23], HBV RNA was suggested as a serum marker for elimination or transcriptional silencing of cccDNA [24]. After long-term NAs treatment, reverse transcription is blocked by NAs, leading to undetectable blood HBV DNA. Since serum HBV RNA becoming the predominant type of HBV virion produced, a positive HBV RNA can be indicative of continued viral transcriptional activity [23]. Several studies researched the value of serum HBV RNA for the safe NAs cessation [17, 23]. Wang et al. [17] found that all EOT serum HBV RNA positive patients (21/21) occurred VR, whereas VR only occurred in 25% (3/12) of EOT serum HBV RNA negative patients during the 24 weeks off treatment (*p* < 0.01). Meanwhile, some studies reported no difference between patients with and without off-treatment VR in the EOT HBV RNA level [25, 26]. The results of this study showed patients without VR had more frequently negative HBV RNA (55.7 vs 87%, *p* < 0.001). When cumulative rates of VR were stratified by EOT HBV RNA, patients with negative EOT HBV RNA had significantly lower rate of VR (39.4 vs 77.5%, *p* < 0.001, Fig. 1C). In the multivariable logistic regression analysis, EOT HBV RNA positive (OR 3.453, 95% CI 1.387–8.597, *p* = 0.008) was found to be independently significant in predicting the risk of VR. All of the above results support the predictive value of HBV RNA for safe NAs cessation in initially HBeAg-positive patients. Meanwhile, automated and high-throughput methods for HBV RNA detection facilitate the expanded use of this marker in clinical practice.

The results of Jung et al. study [22] found that an EOT HBcrAg level  > 3.7 log_10_ IU/mL predicted VR in one year after NAs cessation. The Japan Society of Hepatology divided patients with HBcrAg  < 3 log_10_ U/mL and HBsAg  < 80 IU/mL into low risk group, for which NAs cessation may be considered, with predicted success rate 80–90% [27]. In the present study, patients without VR had lower EOT HBcrAg levels (median, 4 vs 3.5 logU/mL, *p* < 0.001). when cumulative rates of VR were stratified by EOT HBcrAg level, the cumulative incidence of VR was significantly lower in patients with lower HBcrAg levels (< 4 log U/mL) than in those with higher HBcrAg levels (≥ 4 log U/mL) (36.3 vs 74.5%, *p* < 0.001, Fig. 1D). In the multivariable logistic regression analysis, EOT HBcrAg  ≥ 4 logU/mL (OR 3.702, 95% CI 1.614–8.488, *p* = 0.002, Table 3) was found to be independently significant in predicting the risk of VR. All the above results confirmed the value of EOT HBcrAg for safe NAs cessation. However, the role of HBcrAg may be limited by the relative insensitive lower limit of quantitation, i.e. 1 KU/mL in which a large proportion (42.1%) of patients with HBeAg-negative CHB would be tested negative [28].

Quantification of serum HBsAg is pivotal in the management CHB. The results of studies showed that lower EOT HBsAg levels predict off-treatment durability. An EOT HBsAg level  < 100 IU/mL is verified to be good value for NAs cessation in both HBeAg-positive and HBeAg-negative CHB patients [13]. In this study, when cumulative rates of VR were stratified by EOT HBsAg level, the 24 months off-treatment cumulative incidence of VR in EOT HBsAg  < 100 IU/mL was 13.6%, but in EOT HBsAg  < 1000 IU/mL was 36.6%. We also confirmed that the EOT HBsAg level is an independent predictor for safe NAs cessation in HBeAg-positive CHB patients by multivariable logistic regression analysis.

It is known that HBsAg loss is the ultimate goal of CHB treatment, but it is too difficult to achieve. Recently, some studies reported that HBsAg clearance after NAs discontinuation because of the host immune activation [10, 11]. In this study, 24 month rate of HBsAg loss was high and most of these patients had EOT HBsAg level  < 100 IU/mL. For patients with EOT HBsAg level  < 100 IU/mL, a recent study reported a higher 5 year rate of HBsAg loss (47%) [29]. Yao et al. reported the 6 year cumulative rate of HBsAg loss was 61.2% in 74 HBeAg-negative patients [11]. Another study found that rates of HBsAg loss at 3 years after NAs cessation were 9 and 14% in HBeAg-positive and HBeAg-negative patients respectively [6]. Because NAs do not have direct immunomodulatory effect, HBsAg loss is rare during NAs treatment. During the long period of HBV suppression by NA treatment, the host immune mechanisms react effectively to the resumed HBV replication. But the immune system needs to be re-exposed to the replicating virus to occur immune clearance. Therefore, NAs cessation might more often lead to HBsAg loss than maintaining NAs treatment.

Recently more studies focused on combining these serum HBV markers to predict safe NAs cessation [18, 22, 25, 30]. In this study, we successively combined EOT HBsAg and HBV RNA, EOT HBsAg and HBcrAg, EOT HBV RNA and HBcrAg, and EOT HBsAg, HBV RNA and HBcrAg to stratify the off-treatment VR rates. Results showed that patients with EOT HBsAg  < 100 IU/mL plus EOT negative HBV RNA had the lowest 24 month VR rate (5%, Fig. 1E). Meanwhile, the AUROC value of the EOT HBsAg  < 100 IU/mL plus EOT HBV RNA negative was the highest (0.698, *p* < 0.001) to predict VR. Because of the significant high off-treatment VR, NAs cessation is suitable only for a small and selected patients. Based on the above results, we proposed that patients with EOT HBsAg  < 100 IU/mL and EOT negative HBV RNA could try to end of NAs treatment, as lower rate of VR during the 24 month follow-up after NAs cessation.

Liver cirrhosis and HCC development may be a concern after NAs cessation. However, due to the short duration of follow-up after NAs cessation in most studies, the data on the occurrence of cirrhosis and HCC are particularly scarce. In Jeng’s study, they reported 21/691 patients developed HCC at a median of 2.04 (0.18–6.73) years after EOT. The annual incidence, 3 and 6 year cumulative incidence was 0.69, 2 and 4% (0.15, 1 and 1% for noncirrhotic patients, 1.3, 4 and 9% for cirrhotic patients), respectively [11]. The incidence rate was not higher than that during treatment of the patients with or without cirrhosis [31]. For the same reason, too few data to analyse the factors related to progression to liver cirrhosis and HCC in CHB patients after NAs cessation. Long-term follow-up studies are needed to determine the risk of liver cirrhosis and HCC development after NAs cessation.

There are some strengths in our study. First, this is a prospective multicenter cohort with comprehensive data collection, increasing the reliability of the results. Second, most studies only provide results of EOT HBV RNA and HBcrAg, in contrast, we supplied serial results of HBV RNA and HBcrAg at each 3 months after NAs cessation, making the consecutive data for analysis. Third, one important reason for the heterogeneity of studies is the difference in NAs withdrawal criteria. Although few studies used the criteria of Asian-Pacific Association for the Study of the Liver (APASL) [22], this study used the Chinese NAs cessation criteria, which limited longer treatment time and consolidation treatment time, so as to provide more research data for discussing different NAs withdrawal criteria. To date, none of the previous studies investigated the role of the Chinese stopping rule in a CHB cohort. Nonetheless, there are some limitations. First, all patients included were initially HBeAg positive. Whether the study results could apply to initially HBeAg-negative patients need further investigation. Second, since enrolled patients with undetectable HBV DNA, we did not get HBV genotype result. Although prior studies including Asian patients with genotype B or C did not find HBV genotype to be associated with off-treatment relapse [12].

In conclusion, the results of this study suggested NAs cessation is suitable only for a small and selected patients. Serum HBV markers will be helpful for selecting optimal candidates to stop NAs treatment. An EOT HBsAg  < 100 IU/mL and EOT negative HBV RNA identified a patient with low risk of off-therapy rebound, facilitating the risk stratification of off-therapy relapse.

## Abbreviations

ALT: Alanine aminotransferase
cccDNA: Covalently closed circular DNA
CHB: Chronic hepatitis B
CI: Confidence interval
CR: Clinical relapse
EOT: End of treatment
HBcrAg: Hepatitis B core-related antigen
HBeAg: Hepatitis B e antigen
HBV: Hepatitis B virus
HBsAg: Hepatitis B surface antigen
HCC: Hepatocellular carcinoma
NAs: Nucleos(t)ide analogues
OR: Odds ratio
pgRNA: Pregenomic RNA
VR: Virological relapse
